# Reduction in ventral striatal activity when anticipating a reward in depression and schizophrenia: a replicated cross-diagnostic finding

**DOI:** 10.3389/fpsyg.2015.01280

**Published:** 2015-08-26

**Authors:** Gonzalo Arrondo, Nuria Segarra, Antonio Metastasio, Hisham Ziauddeen, Jennifer Spencer, Niels R. Reinders, Robert B. Dudas, Trevor W. Robbins, Paul C. Fletcher, Graham K. Murray

**Affiliations:** ^1^Department of Psychiatry, University of CambridgeCambridge, UK; ^2^Wellcome Trust-MRC Institute of Metabolic ScienceCambridge, UK; ^3^Cambridgeshire and Peterborough NHS Foundation TrustUK; ^4^Psychiatric Liaison Service, Ipswich HospitalNorfolk and Suffolk NHS Foundation Trust, UK; ^5^Department of Psychology, University of CambridgeCambridge, UK; ^6^Behavioural and Clinical Neuroscience Institute, University of CambridgeCambridge, UK

**Keywords:** reward system, ventral striatum, monetary incentive delay, depressive symptoms, research domain framework

## Abstract

In the research domain framework (RDoC), dysfunctional reward expectation has been proposed to be a cross-diagnostic domain in psychiatry, which may contribute to symptoms common to various neuropsychiatric conditions, such as anhedonia or apathy/avolition. We used a modified version of the Monetary Incentive Delay (MID) paradigm to obtain functional MRI images from 22 patients with schizophrenia, 24 with depression and 21 controls. Anhedonia and other symptoms of depression, and overall positive and negative symptomatology were also measured. We hypothesized that the two clinical groups would have a reduced activity in the ventral striatum when anticipating reward (compared to anticipation of a neutral outcome) and that striatal activation would correlate with clinical measures of motivational problems and anhedonia. Results were consistent with the first hypothesis: two clusters in both the left and right ventral striatum were found to differ between the groups in reward anticipation. *Post-hoc* analysis showed that this was due to higher activation in the controls compared to the schizophrenia and the depression groups in the right ventral striatum, with activation differences between depression and controls also seen in the left ventral striatum. No differences were found between the two patient groups, and there were no areas of abnormal cortical activation in either group that survived correction for multiple comparisons. Reduced ventral striatal activity was related to greater anhedonia and overall depressive symptoms in the schizophrenia group, but not in the participants with depression. Findings are discussed in relation to previous literature but overall are supporting evidence of reward system dysfunction across the neuropsychiatric continuum, even if the specific clinical relevance is still not fully understood. We also discuss how the RDoC approach may help to solve some of the replication problems in psychiatric fMRI research.

## Introduction

Current psychiatric diagnostic manuals divide psychopathology into separate diagnostic categories based in the co-occurrence of signs and symptoms rather than on the basis of underlying physiology. However, within each of these categories there is great heterogeneity in the type and severity of symptoms and, similarly, it is common to have overlap in symptoms between the different diagnoses (Lilienfeld, 2014). Recently the Research Domain Criteria (RDoC) framework has been proposed as an alternative framework in order to advance the understanding of mental disorders (Insel et al., 2010). The specific aim of the RDoC project is to increase research that validates new cross-diagnostic dimensions and biological and behavioral measures to carry out better classifications of mental problems. To achieve this improved classification, the RDoC group has proposed a set of five domains or functional systems that are typically affected in psychopathology, and seven units of analysis at which these five constructs can be studied, thus creating a 2-dimensional matrix that can guide research. Additionally, this matrix also includes a further column of “paradigms,” that is, tools that can be used to measure abnormalities in the domains (Cuthbert, 2014a).

Schizophrenia patients, as defined in current diagnostic manuals, typically suffer from symptoms of psychosis (delusions, hallucinations, and disordered behavior, thought, and speech), whereas depression patients are diagnosed on the basis of low mood, anhedonia, and accompanying physical symptoms such as reduced energy. However, psychotic symptoms can appear in the course of a major depressive episode and low mood, blunted affect, alogia, or anhedonia are also characteristic of schizophrenia (Bedwell et al., 2014). Moreover, the boundary between depression and schizophrenia is frequently unclear, and the diagnosis of schizoaffective disorder is often used in cases in which a patient has features of mood disorder and schizophrenia. Additionally, the existence of mild symptoms of psychosis in young people, previously thought to be confer a high risk for schizophrenia, has been shown to be a more general risk factor for different psychiatric disorders including depression (Murray and Jones, 2012; Hui et al., 2013).

At the cognitive-behavioral construct level, disrupted reward processing has been implicated in both schizophrenia and depression. Moreover, it has been suggested that whereas reward receipt may only be subtly affected in both disorders (Cohen and Minor, 2010; Arrondo et al., 2015), the anticipation of reward (Juckel et al., 2006b; Sherdell et al., 2012) and its motivational aspects (e.g., the effort that a subject is willing to make to get it) (Treadway et al., 2012; Barch et al., 2014; Gard et al., 2014) may be markedly dysfunctional in schizophrenia and in depression (for in-depth reviews of the issue see Barch and Dowd, 2010; Kring and Caponigro, 2010; Treadway and Zald, 2011; Der-Avakian and Markou, 2012; Argyropoulos and Nutt, 2013; Kring and Barch, 2014; Whitton et al., 2015). Abnormalities in reward prediction error signaling in the striatum in schizophrenia are also a well-known finding that could be involved in the pathogenesis of psychotic symptoms (Fletcher and Frith, 2009; Ziauddeen and Murray, 2010). Similar changes have also been found in depression (Kumar et al., 2008), and indeed when participants in both patient groups were studied within the same prediction error learning paradigm researchers found brain activation differences between controls and the two groups of patients (Gradin et al., 2011).

Hence, consistent with the RDoC proposal, there appears to be dimensional continuity between schizophrenia and depression with respect to at least some aspects of reward processing. According to the RDoC matrix some of the stated similarities would fit in the Positive Valence systems domain, which is defined as involving “Systems primarily responsible for responses to positive motivational situations or contexts, such as reward seeking, consummatory behavior, and reward/habit learning,” and specifically the Expectancy/Reward Prediction Error component within the Approach Motivation subsystem. The ventral striatum/nucleus accumbens is considered a key area involved in the processing of reward anticipation, and the main neurotransmitter thought to be involved in predicting rewards and learning from them is dopamine. The Monetary Incentive Delay task (MID) is a paradigm that it is well-known to elicit strong striatal activations related to the expectation and salience of rewards (Knutson et al., 2001). It has been widely used both in the healthy population and in patients with neuropsychiatric symptoms, with several results pointing toward both schizophrenia and depression patients having a decreased activity in the striatum when anticipating rewards (Juckel et al., 2006a,b; Nielsen et al., 2012; Stoy et al., 2012). Moreover, reanalyzing data from a set of studies, Hägele and colleagues showed that depression, schizophrenia and alcohol disorders were all associated with reduced activity in the right ventral striatum with this effect correlated with depressive symptoms as measured by the Beck Depression Inventory (Hägele et al., 2015). However, given that patient groups were not matched to each other or to controls in age and gender in the study by Hägele and colleagues we sought to replicate and extend it using a specifically-designed study with matched groups; we also wished to relate neural responses to additional key symptoms, as previous work has indicated that ventral striatum (de)activation could also relate to other symptoms such as anhedonia (Simon et al., 2010; Stoy et al., 2012) and more generally, to positive (Nielsen et al., 2012), and negative (Juckel et al., 2006a; Waltz et al., 2009) symptoms.

In brief, we compared patients with depression and schizophrenia to healthy controls in a Monetary Incentive Delay task. The aim was to further understand how perturbation of the anticipation of reward relates to anhedonia, depression, and overall positive and negative symptoms. Consistent with the results of Hägele and colleagues, we hypothesized that the two clinical groups would have a reduced activity in the ventral striatum when anticipating reward and that the striatal activation would correlate with clinical measures (in a direction such that patients with the least activation would have the more pronounced psychopathology).

## Materials and methods

### Participants

Sixty seven participants were recruited for the study, of whom 22 had a diagnosis of schizophrenia, 24 a diagnosis of major depressive disorder and 21 were healthy volunteers. DSM-IV criteria were used for group classification. Inclusion criteria were to be aged between 18 and 65 and speak English proficiently. Exclusion criteria were any contraindication for entering a MRI scan and history of neurological disorder, physical illness, and alcohol or drug dependence. Participants from both clinical groups had subjective symptoms of anhedonia. Demographic and clinical details of participants are provided in Table 1. Patients with schizophrenia were recruited through psychiatric community services of the Cambridgeshire and Peterborough NHS Foundation Trust. Patients with depression were recruited through psychiatric and psychological community services of the Cambridgeshire and Peterborough NHS Foundation Trust, and through public advertisement. Diagnoses for patients from psychiatric services of the Cambridgeshire and Peterborough NHS Foundation Trust and suitability for the study were confirmed by the review of all available clinical and anamnestic information by each individual's psychiatrist (an experienced psychiatrist with several years of postgraduate experience who had passed the membership examination of the Royal College of Psychiatrists). Diagnoses for patients recruited from psychology services or advertisement were confirmed by a psychiatric interview including PANSS (Kay et al., 1987) and assessment with the Mini-International Psychiatric Inventory (Sheehan et al., 1998) the interview was conducted either by an experienced research psychiatrist with several years of postgraduate experience who had passed the membership examination of the Royal College of Psychiatrists or by a registered clinical psychologist with several years postgraduate experience.

**Table 1:** **Group demographics and clinical characteristics**.

**PARAMETRIC ONE-WAY ANOVA#**										
	**C Mean ± sd (n)**	**D Mean ± sd (n)**	**S Mean ± sd (n)**	**Test statistic (F)**	**p Statistic**	**p Levene**	*****Post-hoc*** pair-wise comparisons**			
							**C vs. D**	**C vs. S**	**D vs. S**	**Summary**
Age#	34.33 ± 10.11 (21)	33.08 ± 9.15 (24)	32.73 ± 7.62 (22)	0.19	0.828	0.294				
Culture Fair (IQ)#	114.24 ± 19.97 (21)	107.08 ± 16.6 (24)	99.38 ± 19.16 (21)	3.38	0.040	0.681	0.486	0.034	0.422	C>S
Education (years)#	14.85 ± 1.93 (20)	13.43 ± 2.21 (23)	13.62 ± 2.11 (21)	2.81	0.068	0.697				
**CHI SQUARE**										
	**C ratio**	**D ratio**	**S ratio**	**Test statistic (X^2^)**	**p Statistic**	**p Levene**	***Post-hoc* pair-wise comparisons**			
							**C vs. D**	**C vs. S**	**D vs. S**	**Summary**
Gender (male/female)	17/4	17/7	19/3	1.71	0.425					
Handedness (right/left)	18/3	22/2	17/5	2.00	0.367					
White-British/other	17/4	20/2	17/5	0.04	0.978					
**KRUSKAL-WALLIS ANOVA**										
	**C Median, IQR (n)**	**D Median, IQR (n)**	**S Median, IQR (n)**	**Test statistic (H)**	**p Statistic**	**p Levene**	***Post-hoc* pair-wise comparisons**			
							**C vs. D**	**C vs. S**	**D vs. S**	**Summary**
BPRS	24, 1 (21)	44, 11.25 (24)	40, 14.5 (22)	42.47	< 0.001		< 0.001	< 0.001	1	S&D>C
BDI	3, 8 (21)	32, 8 (24)	20, 11 (22)	47.37	< 0.001		< 0.001	0.001	0.005	D>S>C
SHAPS	24, 6 (21)	36, 10 (24)	29.5, 8 (22)	25.82	< 0.001		< 0.001	0.012	0.091	D&S>C
TEPS ant	43.5, 8.8 (20)	26.5, 11 (24)	35, 11.5 (22)	22.04	< 0.001		< 0.001	0.048	0.066	C>S>D
TEPS con	37, 8.75 (20)	44, 11.25 (24)	40, 14.5 (22)	16.00	< 0.001		< 0.001	0.012	1	C>S&D
TEPS total	80, 17 (20)	53.5, 21 (24)	62, 18.25 (22)	22.22	< 0.001		< 0.001	0.017	0.159	C&S>D
PANSS +	7, 0 (21)	7, 1	13, 11.5	26.15	< 0.001		0.372	< 0.001	0.001	S>D&C
PANSS −	7, 0 (21)	12.5, 9	14, 6	38.79	< 0.001		< 0.001	< 0.001	0.273	D&S>C
SANS	0, 0 (20)	2, 1	2, 1	42.49	< 0.001		< 0.001	< 0.001	0.495	S&D>C

*Comparisons between groups consisted in parametric One-Way ANOVAs, chi square tests or non-parametric ANOVAs. Post-hoc comparisons were corrected for multiple comparisons. Any significant post-hoc test results for two-group (pair-wise) comparisons (p < 0.05) are also indicated by the use of “greater than” symbols (eg., S&D>C indicates that results in pair-wise comparisons S vs. C and D vs. C were significant). C is the control group, D depression, and S schizophrenia. “p statistic” is the p-value of the omnibus test; “p Levene” is the p-value of Levene's test for the inequality of variances. Standard deviations are denoted as ±sd. IQR stands for interquartile range and n refers to the group sample size for each comparison. BPRS is Brief Psychiatric Rating Scale, BDI is Beck Depression Inventory, SHAPS is Snaith–Hamilton Pleasure Scale, TEPS is the Temporal Experience of Pleasure Scale (ant, anticipatory subscale; con, consummatory subscale), PANSS is Positive and Negative Syndrome Scale (+, positive symptoms; −, negative symptoms), and SANS is Scale for the Assessment of Negative Symptoms. #Results of Kruskal-Wallis ANOVA for these variables can be found in Supplementary Material*.

Thirteen of the depressed participants were taking antidepressant medication: citalopram 30–60 mg daily, mirtazapine 30–45 mg, and venlafaxine 75–225 mg. All patients with schizophrenia were taking atypical antipsychotic medication (specifically clozapine, aripiprazole, risperidone, quetiapine, or olanzapine); two patients were taking a combination of typical and atypical medication. The mean chlorpromazine equivalent dose was 401.24 (*sd* 91.43) mg/day (Kroken et al., 2009). Eight patients with schizophrenia were additionally taking antidepressant medication: citalopram 20–40 mg, fluoxetine 20 mg, mirtazapine 45 mg, venlafaxine 150–225 mg.

The study was conducted at University of Cambridge (Wolfson Brain Imaging Centre and Department of Psychiatry). All participants were evaluated using the following clinical scales: Brief Psychiatric Rating Scale (BPRS, Overall and Gorham, 1962); Positive and Negative Syndrome Scale (PANSS, Kay et al., 1987); Scale for the Assessment of Negative Symptoms Beck Depression Inventory, (SANS, BDI, Beck et al., 1996); Snaith–Hamilton Pleasure Scale (SHAPS, Snaith et al., 1995); and the Temporal Experience of Pleasure scale–TEPS, (Gard et al., 2006). Scales were selected to measure constructs with a possible striatal neural substrate and also according to previous findings of significant correlations with ventral striatum activity during the MID. The Cattell Culture Fair Intelligence Test (CFIT) was used to measure IQ (Cattell et al., 1973).

The study was approved by the Cambridgeshire 3 National Health Service research ethics committee. Written informed consent was obtained from all participants prior to participation.

### fMRI paradigm

**Figure 1:**
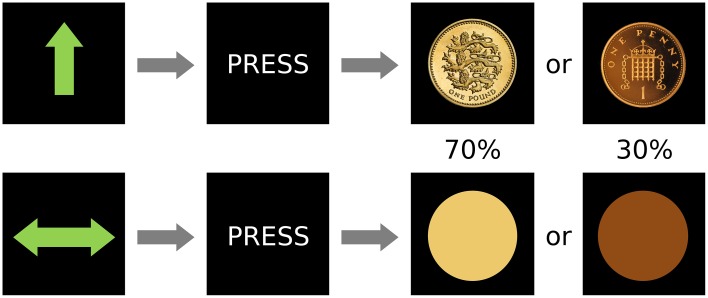
**Design of the paradigm**. There were two conditions, reward (top) and neutral (bottom) that were signaled by an unambiguous cue and were followed by the subject pressing a button and then by two possible outcomes with different frequencies (0.7 or 0.3 probability of occurrence).

The fMRI paradigm was a variation of the Monetary Incentive Delay (MID) task (Figure 1). It used an event-related design in which stimuli served as cues signaling the subsequent outcome. Overall, there were 60 trials in the experiment, which was conducted in a single scanning session. There were two types of cues (after Kirsch et al., 2003): reward cue (an arrow pointing upwards; 30 events) or neutral (a horizontal bar with arrows in both extremes; 30 events), and the participants were instructed to press a button in a rapid manner when requested, after the cues disappeared but before the outcome was known. After a 1–4 s random interval showing a fixation cross, the image of a coin indicated the amount of reward (£1 in 70% of the events and 1 penny in 30%; 21 and 9 events) in the case of the win cue, whereas a yellow or orange circle (70 and 30% of events, respectively; 21 and 9 events) were shown after the neutral cue. Hence, despite our instruction to the participants (which was designed to help engagement with the task), rewards did not depend on the subject's performance while pressing the button. This alteration from the original MID task was intended to reduce the confounds of motor preparation and task-induced anxiety which have been proposed as possible reasons for the previously inconsistent results in depression using the MID (Treadway and Zald, 2011). The inter-trial interval, in which a black screen was shown, lasted between 2 and 6 s. Reward and neutral cues, as well as ensuing outcomes were pseudo-randomly presented. Thus, the design was optimized to detect differences between the two anticipation conditions. Behavioral information obtained from the task included reaction time and responses. Data on response times was lost for 8 participants due to programming problems that did not affect acquisition of other data.

### MRI acquisition and preprocessing

A Siemens Trio Tim 3 T scanner with a 12 channel head coil was used for image acquisition. Functional images were obtained using a Gradient-echo T2^*^-weighted echo planar sequence and consisted in 32 non-contiguous oblique axial planes (in order to minimize signal drop-out in ventral regions, which was especially important according to our hypothesis). Other parameters included relaxation time = 2000 ms; echo time = 30 ms; flip angle = 78; voxel size = 3.14 × 3.14 × 3.75 mm^3^, matrix size 64 × 64; bandwidth 2232 HZ/Px. The structural image was obtained using a high-resolution T1-weighted three-dimensional MP-RAGE sequence.

Imaging preprocessing and analysis was carried out using the FEAT v5.98 (FMRI Expert Analysis Tool) routine within the FSL program (FMRIB's Software Library, www.fmrib.ox.ac.uk/fsl). Functional time-series were sequentially realigned, coregistered to a whole brain echo-planar image and finally to the structural high resolution T1 image, and non-brain components were removed. Functional images were also spatially smoothed using a 6 mm at full width half-maximum (FWHM) Gaussian kernel and frequency filtered (130 s cut off). Images were normalized to the Montreal Neurological Institute (MNI) standard template and the first six volumes were discarded to allow for T1 equilibration effects.

### Statistical analysis

#### Non-imaging data

Non-imaging comparisons were carried out in SPSS 21 (IBM, Armonk, NW, US). Results were considered significant if *p* < 0.05. Normality of all variables was initially evaluated through visual inspection of histograms, whisker plots, and Q-Q plots in the 3 groups.

Results from clinical scales did not follow a normal distribution in at least one of the groups. Hence, differences between groups were tested using Kruskal-Wallis ANOVA. To investigate differences in proportions (gender, handedness, and ethnicity) Chi-square comparisons were carried out. Finally, variables which a-priori were considered to be more likely to meet ANOVA assumptions, and for which the results of the initial inspection was less clear (age, intelligence, and education), were taken to a One-Way ANOVA, residuals saved, and explored through inspection of histograms, whisker plots, and Q-Q plots. In the case of these three variables there was some evidence for a non-fully normal distribution of the residuals. However, since ANOVA is considered to be robust to deviations from normality, parametric results are reported in the main article. Additionally, results from Kruskal-Wallis ANOVAs for these variables can be found in Supplementary Material.

Whenever an ANOVA was significant, we conducted *post-hoc* tests consisting of pair-wise comparisons corrected for multiple testing. In the case of Kruskal-Wallis ANOVA, adjusted significance levels were calculated by multiplying the unadjusted significance values by the number of comparisons with a maximum *p*-value of 1 (SPSS standard method). Gabriel (due to the slightly unequal sample sizes) or Games-Howell procedures were used for One-Way ANOVAs depending on the result of Levene's test on the inequality of variances; the latter was used if the test was significant.

In the case of response times, whisker plots representing data in the reward and neutral condition showed that a participant with depression had a much higher response time in both conditions. However, the difference between the RT in both conditions was within normal parameters (as confirmed by the evaluation of the residuals of the RT differences between conditions), and therefore not likely to influence the results. Nevertheless, we carried out a repeated-measures ANOVA (within subjects effect: Two levels of condition, between subjects effect: group) with and without this participant.

#### Imaging data

We used a single statistical linear regression model with 6 explanatory variables (reward cue, neutral cue; high reward outcome, low reward outcome, and the two neutral outcomes) and their temporal derivatives. Movement parameters from the realignment step were also included in the first-level model.

The a-priori contrast of interest was the anticipation of reward and consisted of the comparison of the BOLD levels during the reward cue and the neutral cue. The reward anticipation contrast uses all cue events in the experiment, with 30 neutral cues and 30 reward cues. Other possible contrasts included the comparison between outcomes, but it was decided not to investigate them at the group level due the reduced number of events that they involved, and because well-predicted rewards often evoke limited brain activity at the time of reward delivery (Berns et al., 2001).

The reward anticipation contrast (reward cue vs. neutral cue) from the first level was taken to the group-level analysis, where it was included in a one sample analysis (control group only, to illustrate this contrast in the healthy population), and One-Way between groups ANOVA (to investigate group differences). Differences were evaluated at the whole brain level and within an a-priori volume of interest mask of the ventral striatum previously used by our group (Bernacer et al., 2013). This region of interest (ROI) included the nucleus accumbens and ventral aspects of the caudate nucleus and putamen (blue regions in **Figure 4**). Comparisons at the whole brain level and within the ROI were cluster-thresholded using a family-wise error (FWE) correction of *p* < 0.05 after a strict initial cluster threshold of *Z*>3 (Woo et al., 2014). Uncorrected results are also displayed in Supplementary Material as part of exploratory analyses that may be of use in future hypothesis generation and meta-analyses, using the same *Z*>3 threshold and a minimum cluster size of 10 across the whole brain.

We extracted the mean parameter estimates for all clusters of differential activation between groups in the imaging ANOVA analysis (using the FSL tool Featquery in the normalized individual images). Then, *post-hoc* pairwise comparisons (two-tailed *t*-tests, equality of variances was not assumed if Levene's test was statistically significant), aimed at exploring the group differences that were driving the significant ANOVA results, were carried out in SPSS. The same *post-hoc* analysis procedure was used for the significant clusters within the ROI comparison of the ventral striatum region.

Activation tables were created using Autoaq (Automatic atlas queries for fsl: http://brainder.org/2012/07/30/automatic-atlas-queries-in-fsl). Number of voxels, maximum voxel *Z*-value (Z max), MNI coordinates of the maximum peak (MAX X,Y,Z), anatomical label of the max peak and significant pairwise *post-hoc t*-test comparisons are reported within tables. Anatomical labels were the most probable location of the highest peak according to the Harvard-Oxford cortical and subcortical structural atlases included in FSL (Desikan et al., 2006). Images were created using MRIcroN (C. Rorden; http://www.mccauslandcenter.sc.edu/mricro/mricron/index.html) and presented in neurological form (right in the image corresponds to the right hemisphere).

A secondary analysis consisted of carrying out Spearman correlations between the mean parameter estimates in the right ventral striatum and clinical symptoms in each of the groups separately. The right ventral striatum was selected as it was the region with reduced activation in schizophrenia and in depression.

## Results

There were no group differences in age, gender, handedness, years of education, or ethnicity. As expected, participants with schizophrenia had a lower IQ and patients had greater psychiatric symptomatology than healthy controls (Table 1, Table S1).

**Figure 2:**
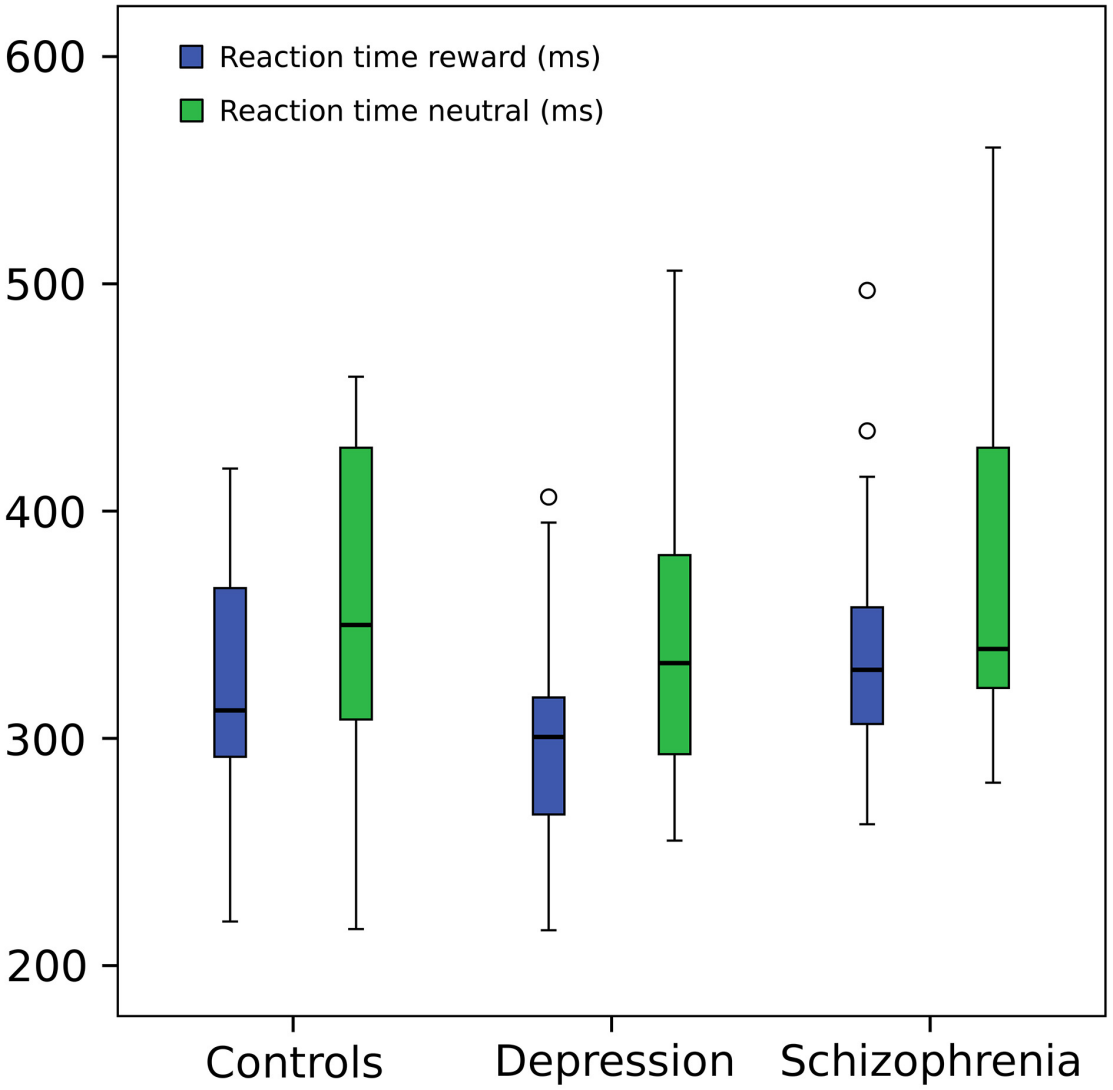
**Reaction time**. Repeated measures ANOVA (within subject factor: condition, between subject factor: group): reaction times in reward trials were shorter (*F* = 36.71, *p* = 0.001) but did not differ between groups (*F* = 0.384, *p* = 0.683). For graphical purposes a subject from the depression group was eliminated from the image due to a much greater mean RT (around 800 ms) in both conditions, but included in the statistical analysis (However, its elimination from the ANOVA did not change significant results). Central line represents the median value (second quartile, Q2) and the box borders indicate the 1st (Q1) and 3rd quartile (Q3). Hence, the total length of the box is the interquartile range (IQR). Whiskers mark last value in the sample located between the 1.5 × IQR below Q1 and 1.5 × IQR above Q3. Circles represent data between the 1.5 and 3 × IQR below Q1 or above Q3.

Response times were shorter in the reward condition (*F* = 36.71, *p*≤0.001) but did not differ between groups (*F* = 0.384, *p* = 0.683). Figure 2 shows whisker plots for the three groups and the two conditions and Table 2 summarizes means and standard deviations. This difference in response time between conditions is characteristic of the paradigm hence indicating that the experimental manipulation was effective in the whole group of participants (Hägele et al., 2015). Results did not change when a subject with slow RTs was taken out (within subject factor *F* = 40.348, *p*≤0.001; between subject factor *F* = 0.624 *p* = 0.540).

**Table 2:** **Response times by group and condition**.

	**C (*n* = 17) Mean ±sd**	**D (*n* = 20) Mean ±sd**	**S (*n* = 22) Mean ±sd**	**p Levene**
RT reward	0.321 ±0.057	0.331 ±0.135	0.342 ±0.058	0.322
RT neutral	0.356 ±0.066	0.367 ±0.126	0.368 ±0.068	0.427

*C is the control group, D depression and S schizophrenia. p Levene is the p-value of Levene's test for the inequality of variances. Standard deviations are denoted as ± sd*.

**Figure 3:**
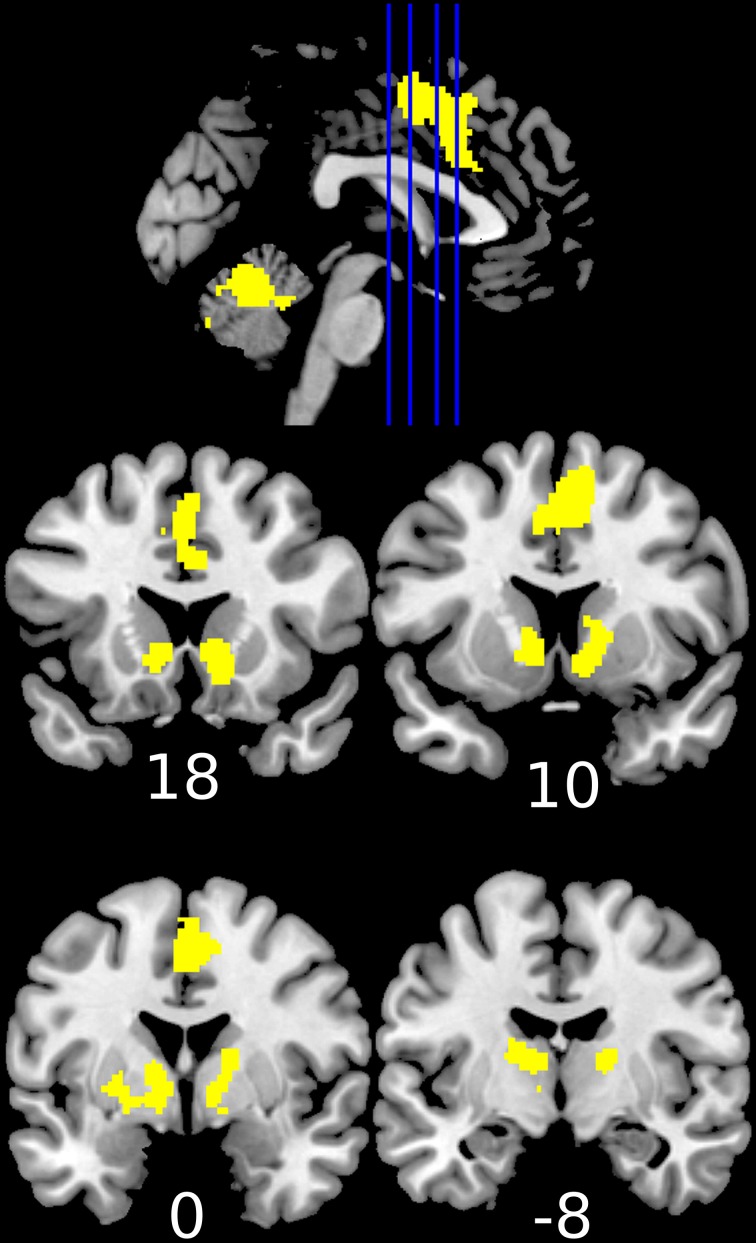
**Increased activation during the anticipation of a reward (reward cue vs. neutral cue) in the healthy controls (1-sample *t*-test of the first level contrast between reward and neutral cues)**. Yellow color indicates voxels within significant clusters corrected at the whole brain level (cluster family wise error corrected *p* < 0.05 after a cluster-inducing primary threshold of *Z*>3). Numbers under slices indicate mm in the MNI coordinate system. The left side of the image represents the left side of the brain.

**Figure 4:**
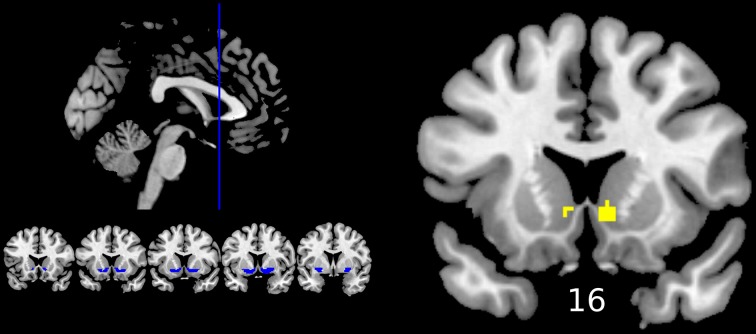
**Differences between groups in reward anticipation (One-Way ANOVA of the first level contrast between reward and neutral cues)**. Yellow color indicates voxels within significant clusters: cluster family wise error corrected *p* < 0.05 within the ventral striatum after a cluster-inducing primary threshold of *Z* > *3*. The region of interest is shown in blue on the left of the image. For improved visualization cluster limits are not circumscribed to our ROI. Differences were driven by greater activations in the healthy controls group compared to both groups of patients (right ventral striatum) or only the depression group (left ventral striatum). Numbers under slices indicate mm in the MNI coordinate system. The left side of the image represents the left side of the brain.

**Figure 5:**
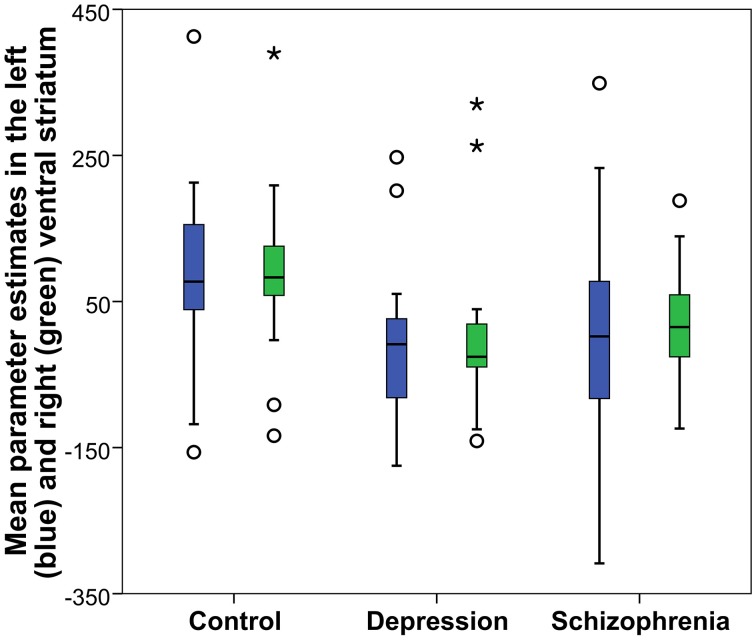
**Reward anticipation mean parameter estimates in the accumbens nucleus: Obtained from the right and left significant clusters (*p* < 0.05 FWE cluster corrected after a cluster-inducing threshold of *Z* > *3* within a ventral striatum ROI) in the between groups One-Way ANOVA of the first level contrast comparing reward and neutral cues**. Parameter estimates are arbitrarily scaled values. Central line represents the median value (second quartile, Q2) and the box borders indicate the 1st (Q1), and 3rd quartile (Q3). Hence, the total length of the box is the interquartile range (IQR). Whiskers mark last value in the sample located between the 1.5 × IQR below Q1 and 1.5 × IQR above Q3. Circles represent data between the 1.5 and 3 × IQR below Q1 or above Q3, whereas asterisks are data further apart from the median.

Reward anticipation (contrast of reward cue vs. neutral cue) in the control group activated areas in the frontal lobe (medial frontal cortex, anterior cingulate cortex), striatum, and thalamus, and cerebellum (FWE cluster corrected across the whole brain *p* < 0.05; see Figure 3 and Table S2). When the three groups were compared using ANOVA, no clusters survived the multiple comparison correction at the whole brain level. However, the ROI analysis in the ventral striatum led to the appearance of two clusters (significant when corrected for multiple comparison) bilaterally in the accumbens nuclei (Figure 4, Table 3). As ANOVA does not indicate the direction of group difference effects, we employed *post-hoc* tests. *Post-hoc* analysis showed activation was significantly greater in the controls than the depressed patients in the right and left accumbens; controls' activation was significantly higher in the right, but not left, accumbens when compared to schizophrenia patients; the two patient groups did not differ from each other (Figure 5 and Table 3).

**Table 3:** **Differences between groups in reward anticipation compared to the anticipation of a neutral outcome (ANOVA F test)**.

**Voxels**	**Z MAX**	**MAX X (mm)**	**MAX Y (mm)**	**MAX Z (mm)**	**Label**	***Post-hoc*** **pair-wise comparisons**			
						**C vs. D**	**C vs. S**	**D vs. S**	**Summary**
17	3.92	8	16	−4	Right Accumbens	*t* = 2.51, *p* = 0.016	*t* = 2.06, *p* = 0.046	*t* = −0.79, *p* = 0.431	C>D&S
1	3.15	−8	16	−4	Left Accumbens	*t* = 2.96, *p* = 0.005	*t* = 1.39, *p* = 0.171	*t* = −0.91, *p* = 0.371	C>D

*Corrected clusters (p < 0.05 FWE after a primary cluster inducing threshold of Z > 3 within the ventral striatum) are displayed. Number of voxels, maximum voxel Z-value (Z max), MNI coordinates of the maximum peak (MAX X,Y,Z), anatomical label of the max peak and post-hoc pair-wise t-tests are reported. C is the control group, D depression, and S schizophrenia. Any significant post-hoc test results for two-group (pair-wise) comparisons (p < 0.05) are also indicated by the use of “greater than” symbols (eg., S&D>C indicates that results in pair-wise comparisons S vs. C and D vs. C were significant)*.

There were two clusters with *Z*-values above 3 and a cluster size greater than 10 in the uncorrected whole-brain analysis (Table S3). One was located in the right accumbens, the other in the frontal pole. A cluster of eight voxels in the left accumbens was the next biggest cluster. The frontal pole result was derived from a reduced activation in the depression group compared to the other two groups.

An analysis of correlations between anhedonia (SHAPS and TEPS scales), depression (BDI), psychiatric symptoms (BPRS), and positive and negative symptoms (PANSS and SANS scales) and the parameter estimates in the right ventral striatal cluster of significant differences in the ANOVA was carried out (Table 4 and Figures S1–S9 in Supplementary Material). A negative correlation between severity of depression and anhedonia symptoms and ventral striatum activity was found in the schizophrenia group. Regarding anhedonia, SHAPS and the total TEPS score were statistically significant, whereas TEPS' subscales showed a trend toward significance (marginal significance). The correlation with SANS was also close to significance. Significant results found in schizophrenia patients did not hold in the other groups and the direction of other correlations, such as the BPRS and PANSS negative symptoms within the depression group, was opposed to that expected.

**Table 4:** **Correlation between symptoms and parameter estimates in the right nucleus accumbens**.

	**C**		**D**		**S**	
	**R**	***p***	**R**	***p***	**R**	***p***
BPRS	0.110	0.664	0.584	0.003	−0.045	0.842
BDI	−0.030	0.898	0.285	0.176	−0.457	0.033
SHAPS	−0.405	0.069	0.163	0.447	−0.483	0.023
TEPS ant	0.237	0314	−0.332	0.113	0.389	0.073
TEPS con	−0.074	0.757	−0.220	0.302	0.396	0.068
TEPS total	0.103	0.665	−0.367	0.078	0.432	0.045
PANSS +	0.111	0.633	0.304	0.149	−0.046	0.840
PANSS −	0.000	1.000	0.471	0.020	−0.300	0.174
SANS	–	–	0.227	0.286	−0.401	0.065

*C is the control group, D depression, and S schizophrenia. Parameter estimates correspond to the mean of the parameter estimates in the clusters of differential activation between groups in the accumbens (obtained from the right significant cluster in the ANOVA comparing reward anticipation activity between groups). R is the Spearman correlation between the measures. BPRS is Brief Psychiatric Rating Scale, BDI is Beck Depression Inventory, TEPS is the Temporal Experience of Pleasure Scale (ant, anticipatory subscale; con, consummatory subscale), SHAPS is Snaith–Hamilton Pleasure Scale, PANSS is Positive and Negative Syndrome Scale (+, positive symptoms; −, negative symtoms), and SANS is Scale for the Assessment of Negative Symptoms*.

## Discussion

We used a Monetary Incentive Delay (MID) paradigm in a single experiment designed to examine activation during reward anticipation in controls, schizophrenia, and depression. Both clinical groups had a reduction in right ventral striatal activity when anticipating rewards as predicted; results in the left ventral striatum were reduced in depression but not definitively reduced in schizophrenia. We were not able to find a clear correlation between striatal activation and clinical symptoms of depression or anhedonia in the depression group, but such a correlation was present in the schizophrenia group. The design of the study is consistent with the Research Domain Criteria (RDoC) project: one of the designs that the RDoC framework has proposed is to include participants with disorders from different sections of the DSM/ICD diagnostic manuals with the aim of exploring an abnormal neurobehavioral construct to further understand its pathological mechanisms (Cuthbert, 2014a). The rationale underlying the study was the fact that neurocognitive domains of motivation and reward processing have been proposed to be abnormal in both disorders, and such abnormalities may be at the root of the some of the common features between schizophrenia and depression.

Our results of reduced right ventral striatal activity during reward anticipation in both depression and schizophrenia can be considered a replication of the work of Hägele et al. which was recently published (2015). The combined evidence of both studies suggests that reduced BOLD signaling in the right nucleus accumbens is indeed a hallmark of pathological reward anticipation. The results in the left accumbens are more equivocal; unlike Hägele and colleagues, we demonstrated reduced left accumbens activation in depression, but similar to Hägele and colleagues we failed to demonstrate a conclusive abnormality here in schizophrenia. Further study will be required to investigate whether there is any fundamental pathological importance in this small laterality effect or whether it relates to more trivial issues such as the precise sensitivity of the paradigm.

In the case of schizophrenia, our findings are not new, as previous studies had also found decreased activity in the basal ganglia and specifically in the ventral striatum (Nielsen et al., 2012), although other smaller studies did not find such differences (Walter et al., 2009; Waltz et al., 2010). On the other hand, results in the MID paradigm with depression patients have been surprisingly inconclusive. For example, the first study using the MID in depression (*n* = 14 patients and 12 controls) showed no evidence of abnormality in the basal ganglia (Knutson et al., 2008). Our study and the work by Hägele et al. are among the ones with the biggest sample sizes and when taken into account in a combined way strongly suggest that abnormalities in the reward circuit are not limited to patients with a diagnosis of schizophrenia, but also relate to patients with depression symptoms. Other data such as a meta-analysis on reward and depression (Zhang et al., 2013) and a study by Pizzagalli et al. (2009), indicate that reduced basal ganglia activations may not only (or even primarily) be located in the ventral striatum, but could also involve other subcortical regions such as the caudate head or the posterior putamen. We were not able to confirm the results of the meta-analysis by Zhang and colleagues suggesting frontal and anterior cingulate over-activation in depression during reward anticipation.

One reason that has been put forward to account for the discrepancies between MID depression studies has been that reward is usually contingent on a speeded performance on the MID, which might be influencing some patients through a stress related, possibly dopaminergic, response (Treadway and Zald, 2011); these authors argue that the necessity to respond rapidly may enhance activation particularly in anxious patients, whose motivation may relate to hypersensitivity to perceived failure. To deal with this potential confound we modified the original task so reward did not depend on the speed of the response. This change may be related to the finding of reduced accumbens activation, which has also been shown in other experiments in which a speeded motor response was not involved (Smoski et al., 2009). It must be noted however that the paradigm used in the studies reported by Hägele et al. (2015) was closer to the original design of the MID and included the necessity of a fast response to obtain the reward. Similarly, we did not include a loss condition in our design, as it has been proposed that the strongest activations in healthy controls and when comparing them to patients come from contrasting the gain and neutral cues (Hägele et al., 2015) Hence, our design may be suitable and sensitive for psychiatric research when the main objective is to study reward processing. Although reward was delivered irrespective of the speed of button pressing, response time in the reward condition was shorter. Valence effects on response time can occur irrespective of a direct consequence of speeded responses (O'doherty et al., 2004; Pessiglione et al., 2006; Murray et al., 2008b). The phenomenon has been termed “reinforcement related speeding” (Cools et al., 2005; Murray et al., 2008a), and it is thought that a potential reward leads to enhanced motivation and hence faster responding (Crespi, 1942).

Whilst the right accumbens was a site of common underactivation in both patient groups compared to controls, no differences between the two patient groups were found. This result indicates that the two groups of psychiatric participants may be more similar to each other than when compared to healthy controls, which would be in accordance with the RDoC perspective of a common abnormal domain. Comparisons between patient groups were not reported in Hägele et al., but a qualitative analysis of their plotted results show them to be in line with ours; the two patient groups had a similar activity reduction in the striatum. Regarding the effects of the medications for psychosis, it is a limitation that all of the schizophrenia patients were taking antipsychotic medication. Thus, we cannot exclude the possibility that the results may in part be secondary to medication effects. However, we note that as a previous study from the Berlin group found that the striatal deactivation normalized when changing from typical to atypical antipsychotics (Schlagenhauf et al., 2008), and as all of our schizophrenia patients were taking atypical antipsychotic medication, it is unlikely that the results are solely due to medication. Nevertheless, it will be important, albeit challenging, to study medication free samples in future research.

The second hypothesis was that the BOLD signal in the striatum would negatively correlate with clinical symptoms of depression and anhedonia. Previous studies have only reported one or two clinical measures per study, but clinical constructs that correlated with the activity of the ventral striatum have included depression (Hägele et al., 2015) anhedonia and apathy (Simon et al., 2010; Stoy et al., 2012), positive symptoms (Nielsen et al., 2012) or negative (Juckel et al., 2006a; Waltz et al., 2009) symptoms, and severity of overall psychiatric symptoms measured by the BPRS (Waltz et al., 2009). In accordance with our cross-diagnostic approach and the aim of further investigating the mechanisms of abnormal reward processing, we decided to include a broad range of clinical measures that encompassed most of the constructs previously reported to correlate with striatal activity in the MID. Results in this regard were mixed. On the one hand we were able to replicate Hägele's et al. results of reduced activity in the right accumbens nucleus of those schizophrenia participants with more depressive symptoms. We also extend the results of Hägele and colleagues to relationships between less activity and more anhedonia in schizophrenia, as measured by the TEPS and SHAPS scales. A limitation of our work is that we did not correct our correlation analyses for multiple comparisons, and considering the modest effect sizes observed, those significant correlations we do find may be vulnerable to Type I error. In contrast to results from Hägele et al., neither the BDI nor any of the other measures were associated with ventral striatal activation in both patient groups. However, the lack of brain-symptom associations demonstrated in the depression group, and the control group (as some of the scales were designed to assess patients only), cannot be considered as evidence of absence of brain-symptom relations. Some correlations such as the significant positive relationship between activation and the BPRS and PANSS (negative symptoms subscale) scales in the depression group were counterintuitive (and not maintained across groups). This may reflect a chance finding, or, as Treadway and Zald (2011) have speculated, the activation elicited by the MID task may be a composite of activation associated with reward anticipation and anticipatory anxiety about potential failure. We attempted to address this possibility by our modification of the task to dissociate reinforcement from performance but it remains possible that, especially in depression, some symptoms may relate to striatal overactivity (leading to greater activation when more psychopathology) and other symptoms may relate to underactivity. The relationship between psychopathology and striatal reward processing activation may be complex, and it is possible that striatal (dys)function may contribute to symptom expression only through interactions with other regional dysfunction and other psychological processes. The concept of dysfunction of one psychological process in one brain area leading to expression of one symptom has the attraction of being a testable hypothesis but is necessarily an oversimplification.

An important challenge in assessing brain-symptom relationships is accurate symptom measurement. This is especially challenging when experts disagree about what constitutes a particular symptom. Anhedonia is defined in the DSM-IV-TR as a loss of interest or pleasure (American Psychiatric Association, 2000), which arguably reflects the consensus use of the term over the past 100 or so years (e.g., Myerson, 1922; see also Berrios, 1996). However, the DSM-5 contains a new definition within the schizophrenia (not depression) chapter, “the decreased ability to experience pleasure from positive stimuli or a degradation in the recollection of pleasure previously experienced,” and arguments continue as to whether a broad or narrow use of the term is more helpful (recently discussed by Treadway and Zald, 2011; Der-Avakian and Markou, 2012; Romer Thomsen et al., 2015). Consensus appears to be building that in depression and schizophrenia, anticipatory, and motivational aspects of reward are more compromised than consummatory (reward receipt) aspects, possibly related to dopaminergic abnormalities in both conditions (Argyropoulos and Nutt, 2013; Kring and Barch, 2014; Whitton et al., 2015); though see a recent study (Gard et al., 2014) documenting enhanced anticipation of pleasure in schizophrenia). However, it remains possible that this relative consensus may in part reflect the methods that have been recently used to investigate the issue. Variants of the monetary incentive delay task as we used here may be more sensitive to reward anticipation effects than reward delivery effects, as well-predicted rewards tend to evoke less strong brain responses than surprising rewards (e.g., Berns et al., 2001). In addition, given the limited temporal resolution of fMRI it can be hard to dissociate anticipatory and consummatory aspects of reward. Furthermore, most fMRI patient studies have, as we did, used monetary rewards, but processing of primary and secondary rewards may differ in important respects (Sescousse et al., 2013).

### Strengths and limitations

As noted, our work is similar to existing research, such as that of Hägele and colleagues. However, there are differences between the two studies. Our study was at higher field strength (we used a 3 Tesla magnet vs. a 1.5 Tesla of Hägele and all). Our study uses a matched control group whereas Hägele and colleagues, because theirs is a retrospective synthesis and re-analysis of previously published separate works, use a control group that is not matched to their patients in the basic features of age and gender. Our study uses different (slightly simpler) stimuli to Hägele and colleagues and, while both studies require a button press, in ours, the reinforcement is not actually contingent on the button press reaction time (as discussed above, this was suggested by Treadway and Zald (2011) as being advantageous in reducing anticipatory anxiety). Our study includes a more detailed assessment of psychopathology (with the limitations that, as previously mentioned, when utilizing the psychopathology for correlation analyses we did not correct for multiple correlations, and that our sample size is modest for correlation analysis).

## Conclusion

In summary, while a reduced activation in the ventral striatum when anticipating rewards is a common endophenotype in psychopathology, the mechanisms underpinning this finding and related symptoms are not completely clear. It will be crucial to further pinpoint the clinical relevance of this finding but it will require further studies and replications. Although some evidence from both the previous literature and our work points toward negative or depressive symptoms being more related to the reported finding, they require further confirmation. The future of this line of research fits nicely within the specifications laid out by the RDoC project, although it also faces similar challenges, such as the measurement error, and the biological and psychometric limitations of proposed endophenotypes and their relationship to behavior (Cuthbert, 2014b; Lilienfeld, 2014; Weinberger and Goldberg, 2014). Upcoming studies on reward and psychopathology will have to use bigger sample sizes and a broader range of clinical measurements in order to be able to obtain a compelling evidence of the relationship between brain activation and everyday behavior. As shown in our work, future studies could benefit from including participants with a range of diagnoses. This aim can be best achieved by a large-scale collaboration across different research groups. Moreover, the wide use of the MID task makes it a good candidate measure for such collaboration, although a common “official” version would be important. Our results indicate that a paradigm that does not base reward on performance might be better fitted for research with stress-prone participants.

Our overall findings are further evidence of reward system dysfunction across the neuropsychiatric continuum, even if the specific clinical relevance is still not fully understood. Studies on this line of fruitful research could provide new insights on the cross-diagnostic mechanisms of psychopathological symptoms, especially if conducted in a way that minimizes the challenges posed to the Research Domain Criteria approach.

## Conflict of interest statement

Trevor W. Robbins has received research support from or served as a consultant to Cambridge Cognition, Eli Lilly, GlaxoSmithKline, and Lundbeck. Hisham Ziauddeen has been jointly funded by the Wellcome Trust and GlaxoSmithKline on the Translational Medicine and Therapeutics programme. Paul C. Fletcher has received funds from GlaxoSmithKline for consultation services and from Astra Zeneca for a lecture. Gonzalo Arrondo, Nuria Segarra, Antonio Metastasio, Jennifer Spencer, Robert B. Dudas, and Graham K. Murray and Niels R. Reinders have no financial interests.
