# Supplementary material for: Reduction in ventral striatal activity when anticipating a reward in depression and schizophrenia: a replicated cross-diagnostic finding
Source: Front Psychol. 2015 Aug 26;6:1280. doi: 10.3389/fpsyg.2015.01280 (PMC4549553; doi:10.3389/fpsyg.2015.01280)

Supplementary Material

Reduction in ventral striatal activity when anticipating a reward in depression and schizophrenia: a robust cross-diagnostic finding

Gonzalo Arrondo[1], Nuria Segarra[1], Antonio Metastasio[1], Hisham Ziauddeen[145], Jennifer Spencer[15], Niels R Reinders[1], Robert B. Dudas[156], Trevor W. Robbins[23], Paul C. Fletcher[1345], Graham K. Murray*[135]

1-Department of Psychiatry, University of Cambridge, Cambridge, United Kingdom
2-Department of Psychology, University of Cambridge, Cambridge, United Kingdom
3-Behavioural and Clinical Neuroscience Institute, University of Cambridge, Cambridge, United Kingdom
4-Wellcome Trust-MRC Institute of Metabolic Science, Cambridge, United Kingdom
5-Cambridgeshire and Peterborough NHS Foundation Trust, United Kingdom
6-Psychiatric Liaison Service, Ipswich Hospital, Norfolk and Suffolk NHS Foundation Trust, United Kingdom

***Correspondence:** Dr. Graham K. Murray, Department of Psychiatry, University of Cambridge, Box 189Addenbrooke's Hospital, Cambridge, CB2 0QQ, UK, Telephone: +44 1233 769499 – Fax +44 1223 336 581, gm285@cam.ac.uk

Index of contents

Table S1. Non-parametric ANOVAs and of group demographics.

Table S2. Increased activation during the anticipation of a reward compared to a neutral stimulus in the healthy controls (1-sample t-test of the first level contrast between reward and neutral cues)

Table S3. Differences between groups in reward anticipation compared to the anticipation of a neutral outcome (ANOVA F test, uncorrected clusters).

Supplementary Figure S1. Mean parameter estimates in rVST and SHAPS

Supplementary Figure S2. Mean parameter estimates in rVST and SANS

Supplementary Figure S3. Mean parameter estimates in rVST and PANSS positive

Supplementary Figure S4. Mean parameter estimates in rVST and PANSS negative

Supplementary Figure S5. Mean parameter estimates in rVST and BPRS

Supplementary Figure S6. Mean parameter estimates in rVST and BDI

Supplementary Figure S7. Mean parameter estimates in RVST and TEPS total

Supplementary Figure S8. Mean parameter estimates in RVST and TEPS total

Supplementary Figure S9. Mean parameter estimates in RVST and TEPS total

Table S1. Non-parametric ANOVAs and of group demographics. Post-hoc comparisons were corrected for multiple comparisons. Any significant post-hoc test results for two-group (pair-wise) comparisons (p<0.05) are also indicated by the use of "greater than" symbols (eg S&D>C indicates that results in pair-wise comparisons S vs. C and D vs. C were significant). C is the control group, D depression and S schizophrenia. p statistic is the p-value of the omnibus test, p Levene is the p-value of Levene's test for the inequality of variances. IQR stands for interquartile range and n refers to the group sample size for each comparison.

PARAMETRIC 1-WAY ANOVA#

	**C** Median, IQR (n)	**D** Median, IQR (n)	**S** Median, IQR (n)	**Test statistic (H)**	**p statistic**	**Post-hoc pair-wise comparisons**			
						**C vs. D**	**C vs. S**	**D vs. S**	**Summary**
**Age**	34, 20 (21)	32.5, 14 (24)	31.5, 13 (22)	0.312	0.856				
**Culture Fair (IQ)**	109, 28 (21)	99.5, 25 (24)	92, 31 (21)	9.174	0.010	1	0.009	0.108	C>S
**Education (years)**	16, 3 (20)	13, 5 (23)	13, 5 (21)	4.85	0.089				

Table S2. Increased activation during the anticipation of a reward compared to a neutral stimulus in the healthy controls (1-sample t-test of the first level contrast between reward and neutral cues). Corrected (p<0.05 FWE after a cluster primary inducing threshold of Z>3 at the whole brain level; showed in bold fonts) and uncorrected clusters (Z>3 at the whole brain level and a cluster size greater than 10) are displayed. Number of voxels, maximum voxel Z value (Z max), MNI coordinates of the maximum peak (MAX X,Y,Z) and anatomical label of the max peak are reported

Voxels	Z MAX	MAX X (mm)	MAX Y (mm)	MAX Z (mm)	Label
**Significant clusters after multiple comparison correction**					
**683**	**3.89**	**-2**	**-56**	**-18**	**No label found**
**678**	**4.07**	**10**	**10**	**56**	**Superior Frontal Gyrus**
**493**	**4.13**	**-14**	**-8**	**12**	**Left Thalamus**
**456**	**4.24**	**10**	**16**	**-4**	**Right Accumbens**
**Significant clusters**					
102	3.66	32	32	-6	Frontal Orbital Cortex
82	3.72	-26	24	-4	Insular Cortex
42	3.73	-30	-22	-4	Left Putamen
34	3.56	-38	42	-4	Frontal Pole
32	3.29	24	-68	-26	No label found
29	3.52	-12	-18	-14	No label found
28	3.56	46	0	44	Precentral Gyrus
26	3.53	26	-70	-56	No label found
25	3.35	36	0	48	Middle Frontal Gyrus
25	3.54	50	-42	16	Supramarginal Gyrus, posterior division
25	3.43	24	52	0	Frontal Pole
22	3.38	-32	-40	34	Supramarginal Gyrus, anterior division
21	3.48	-34	-8	42	Precentral Gyrus
21	3.61	34	58	-10	Frontal Pole
20	3.39	-40	-58	-20	Temporal Occipital Fusiform Cortex
19	3.37	8	-74	-36	No label found
17	3.59	-4	14	20	Cingulate Gyrus, anterior division
15	3.36	20	-12	-10	Right Amygdala
14	3.41	50	18	-8	Temporal Pole
14	3.41	-32	58	-10	Frontal Pole
13	3.31	-40	-4	54	Precentral Gyrus
13	3.36	26	-58	-22	No label found
12	3.29	34	34	20	Frontal Pole
11	3.32	-28	-4	42	Precentral Gyrus
11	3.25	2	36	30	Paracingulate Gyrus

Table S3. Differences between groups in reward anticipation compared to the anticipation of a neutral outcome (ANOVA F test, uncorrected clusters). Z>3 at the whole brain level and cluster size above 10 are displayed. Number of voxels, maximum voxel Z value (Z max), MNI coordinates of the maximum peak (MAX X,Y,Z), anatomical label of the max peak and post-hoc pair-wise t-tests are reported. C is the control group, D depression and S schizophrenia.
Any significant post-hoc test results for two-group (pair-wise) comparisons (p<0.05) are also indicated by the use of "greater than" symbols (eg S&D>C indicates that results in pair-wise comparisons S vs. C and D vs. C were significant).

**Voxels**	**Z MAX**	**MAX X (mm)**	**MAX Y (mm)**	**MAX Z (mm)**	**Label**	**Post-hoc pair-wise comparisons**			
						**C vs. D**	**C vs. S**	**D vs. S**	**Summary**
54	3.92	8	16	-4	Right Accumbens	t=2.80, p=0.007	t=2.06, p=0.021	t=-0.78, p=0.441	C>D&S
11	3.67	34	48	-10	Frontal Pole	t=5.51, p=0.001[#]	t=0.58, p=0.568	t=-3.11, p=0.003	C&S>D
8[##]	3.22	-8	18	-4	Left Accumbens	t=3.03, p=0.004	t=1.70, p=0.097	t=-0.82, p=0.414	C>D

Equal variances were not assumed as Levene's test was statistically significant.
Cluster was included despite its 8 voxel size for information purposes because the cluster is significant when correcting for multiple comparisons within the volume of interest of the ventral striatum.

Supplementary Figure S1. Mean parameter estimates in rVST and SHAPS: Mean parameter estimates (arbitrary scale) in the right ventral striatum (significant cluster in the ANOVA comparing reward anticipation activity between groups) and clinical symptoms. Blue, green and red circles represent healthy controls, participants with depression and participants with schizophrenia respectively. SHAPS is Snaith–Hamilton Pleasure Scale.

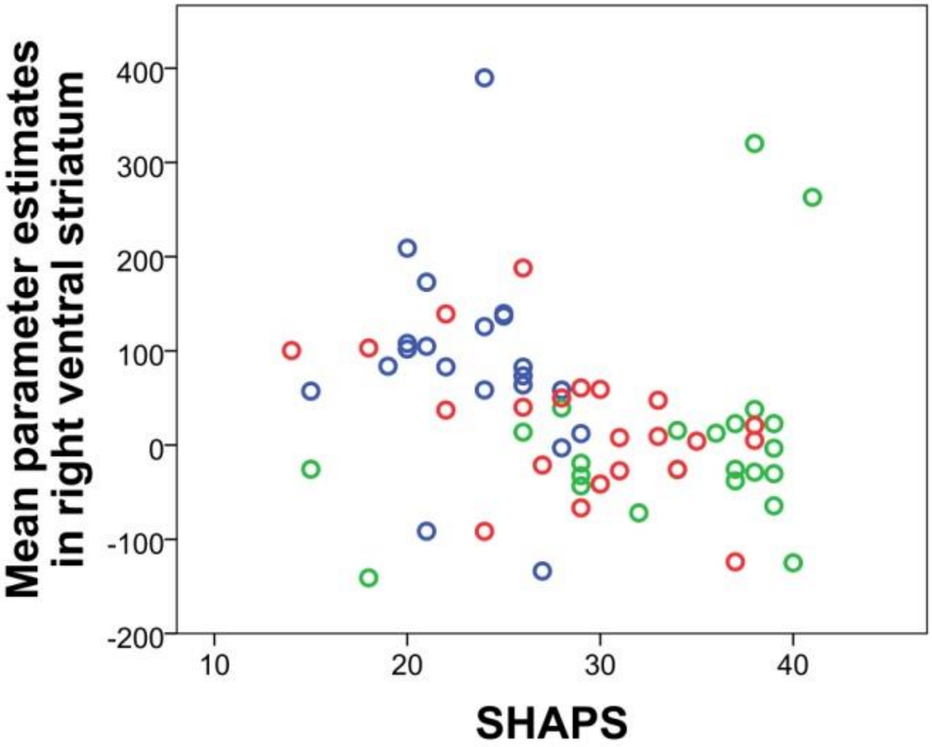

Supplementary Figure S2. Mean parameter estimates in rVST and SANS: Mean parameter estimates (arbitrary scale) in the right ventral striatum (significant cluster in the ANOVA comparing reward anticipation activity between groups) and clinical symptoms. Blue, green and red circles represent healthy controls, participants with depression and participants with schizophrenia respectively. SANS is Scale for the Assessment of Negative Symptoms.

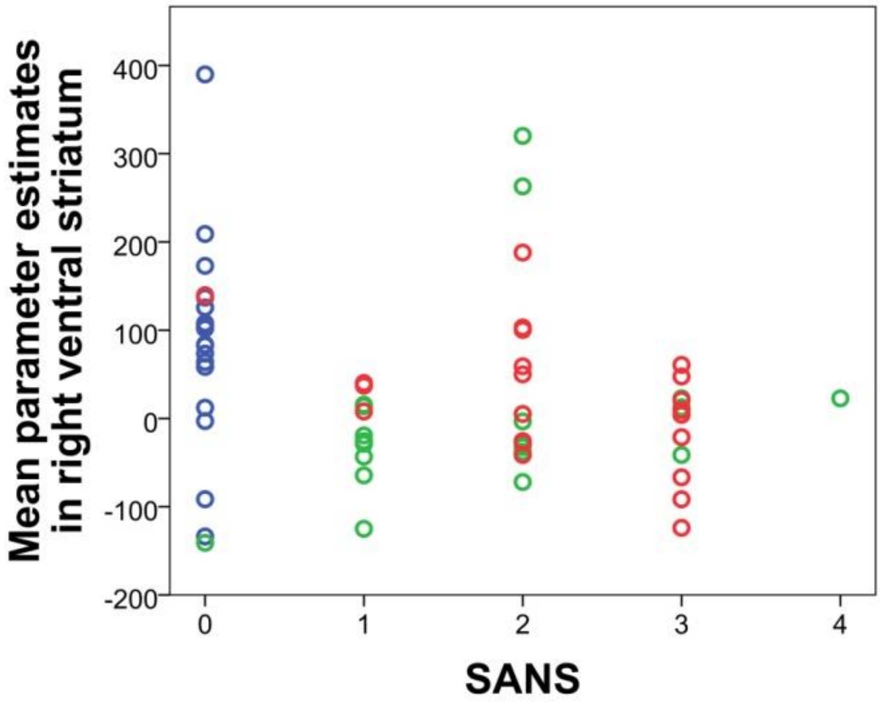

Supplementary Figure S3. Mean parameter estimates in rVST and PANSS positive: Mean parameter estimates (arbitrary scale) in the right ventral striatum (significant cluster in the ANOVA comparing reward anticipation activity between groups) and clinical symptoms. Blue, green and red circles represent healthy controls, participants with depression and participants with schizophrenia respectively. PANSS is Positive and Negative Syndrome Scale; positive subscale.

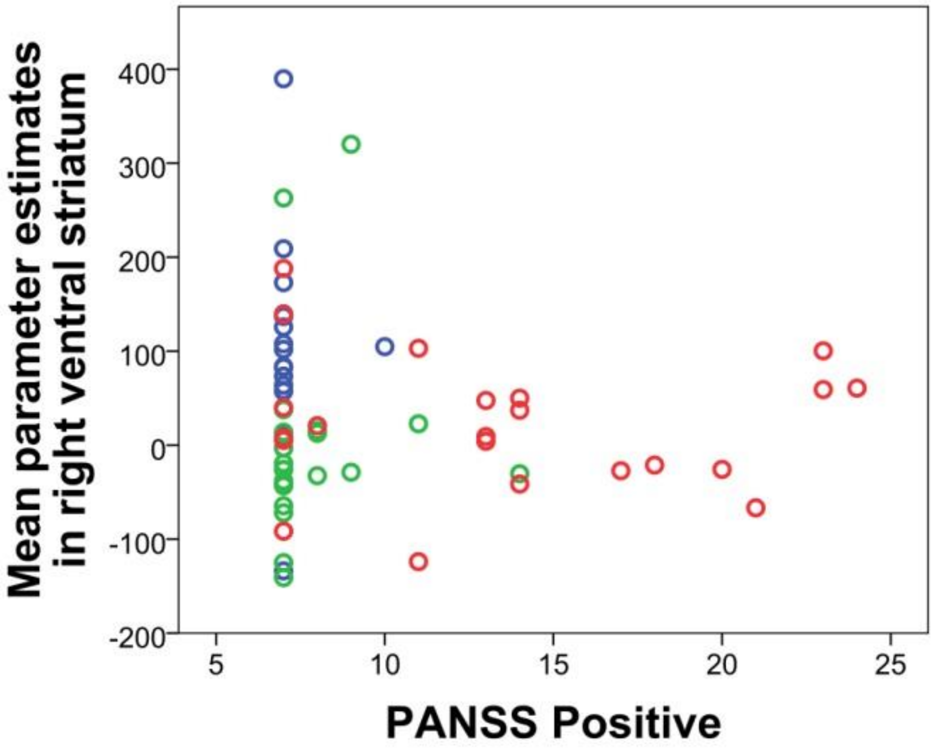

Supplementary Figure S4. Mean parameter estimates in rVST and PANSS negative: Mean parameter estimates (arbitrary scale) in the right ventral striatum (significant cluster in the ANOVA comparing reward anticipation activity between groups) and clinical symptoms. Blue, green and red circles represent healthy controls, participants with depression and participants with schizophrenia respectively. PANSS is Positive and Negative Syndrome Scale; negative subscale.

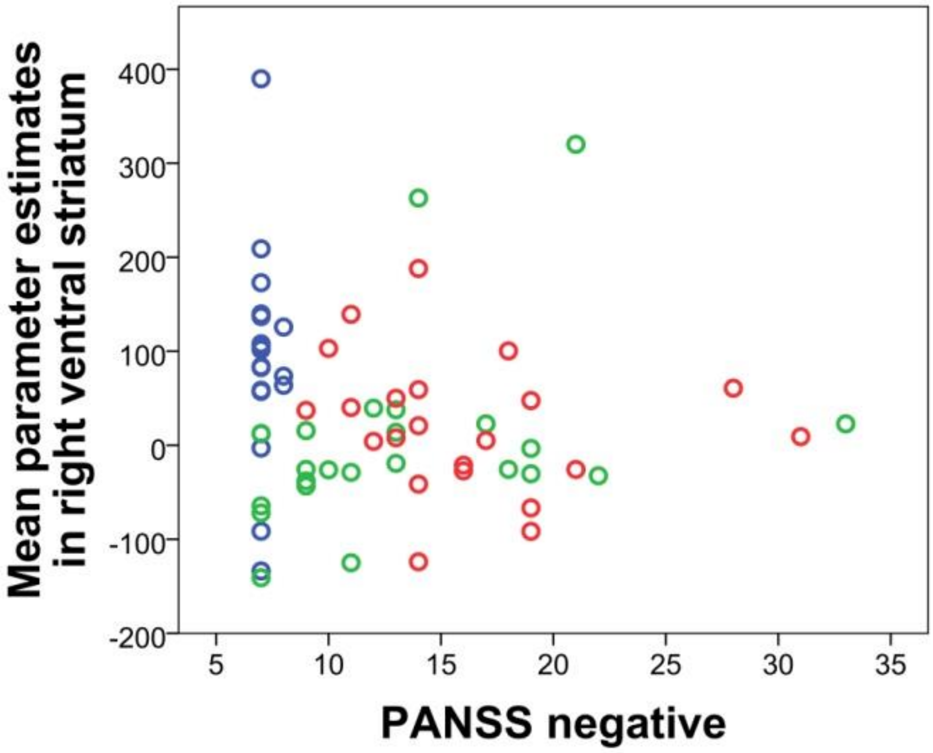

Supplementary Figure S5. Mean parameter estimates in rVST and BPRS: Mean parameter estimates (arbitrary scale) in the right ventral striatum (significant cluster in the ANOVA comparing reward anticipation activity between groups) and clinical symptoms. Blue, green and red circles represent healthy controls, participants with depression and participants with schizophrenia respectively. BPRS is the Brief Psychiatric Rating Scale.

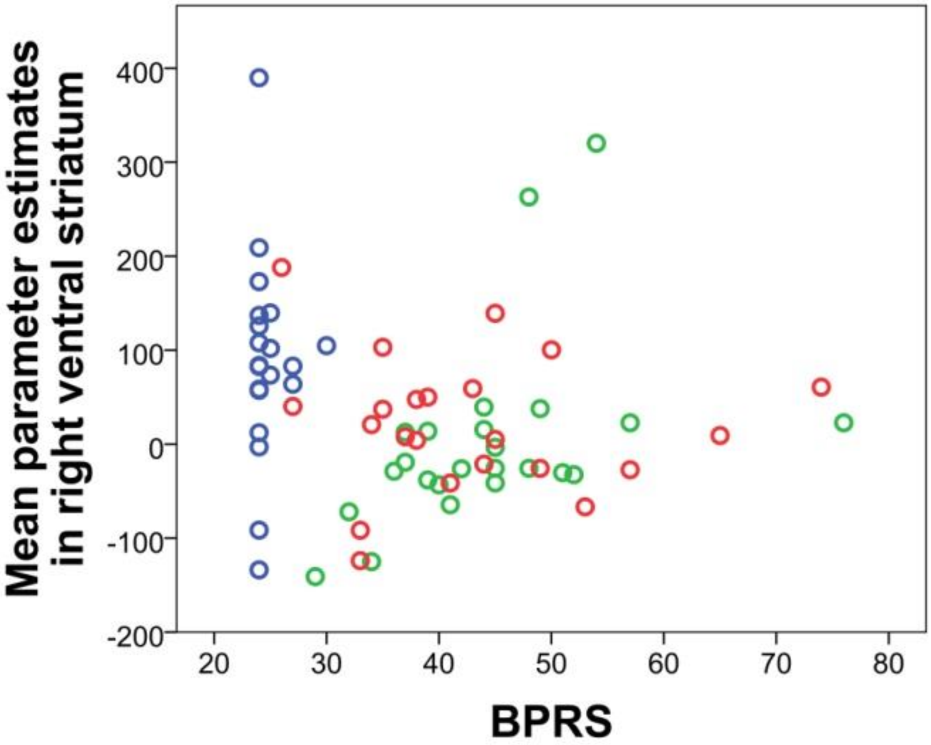

Supplementary Figure S6. Mean parameter estimates in rVST and BDI: Mean parameter estimates (arbitrary scale) in the right ventral striatum (significant cluster in the ANOVA comparing reward anticipation activity between groups) and clinical symptoms. Blue, green and red circles represent healthy controls, participants with depression and participants with schizophrenia respectively. BDI is Beck Depression Inventory.

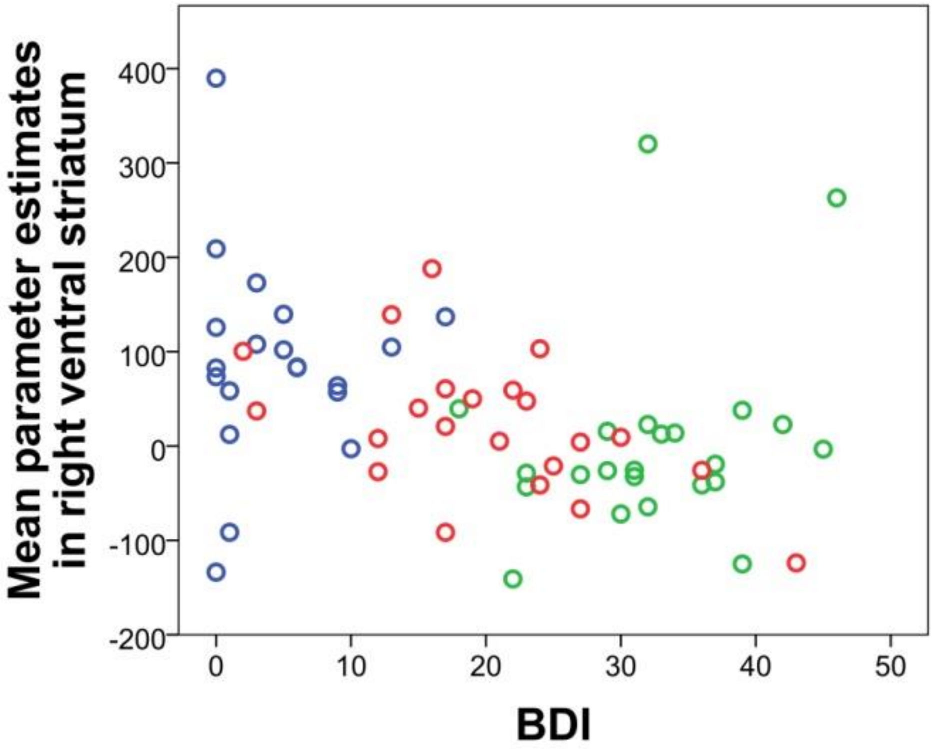

Supplementary Figure S7. Mean parameter estimates in RVST and TEPS total: Mean parameter estimates (arbitrary scale) in the right ventral striatum (significant cluster in the ANOVA comparing reward anticipation activity between groups) and clinical symptoms. Blue, green and red circles represent healthy controls, participants with depression and participants with schizophrenia respectively. TEPS is the Temporal Experience of Pleasure Scale; total result.

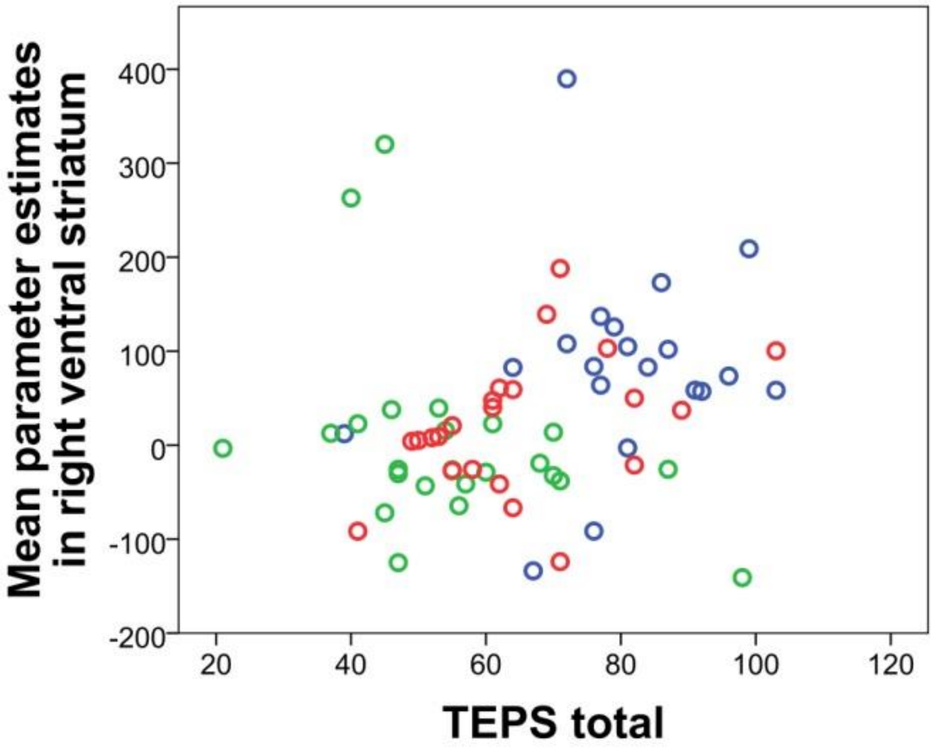

Supplementary Figure S8. Mean parameter estimates in RVST and TEPS consummatory subscale: Mean parameter estimates (arbitrary scale) in the right ventral striatum (significant cluster in the ANOVA comparing reward anticipation activity between groups) and clinical symptoms. Blue, green and red circles represent healthy controls, participants with depression and participants with schizophrenia respectively. TEPS is the Temporal Experience of Pleasure Scale; consummatory subscale.

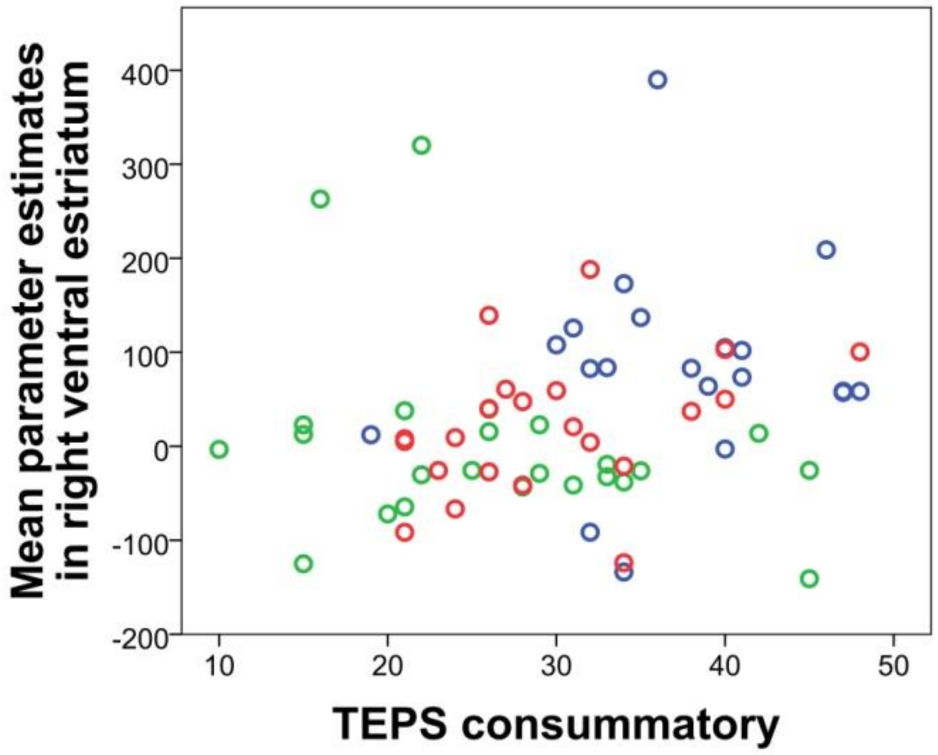

Supplementary Figure S9. Mean parameter estimates in RVST and TEPS anticipatory subscale: Mean parameter estimates (arbitrary scale) in the right ventral striatum (significant cluster in the ANOVA comparing reward anticipation activity between groups) and clinical symptoms. Blue, green and red circles represent healthy controls, participants with depression and participants with schizophrenia respectively. TEPS is the Temporal Experience of Pleasure Scale; anticipatory subscale.

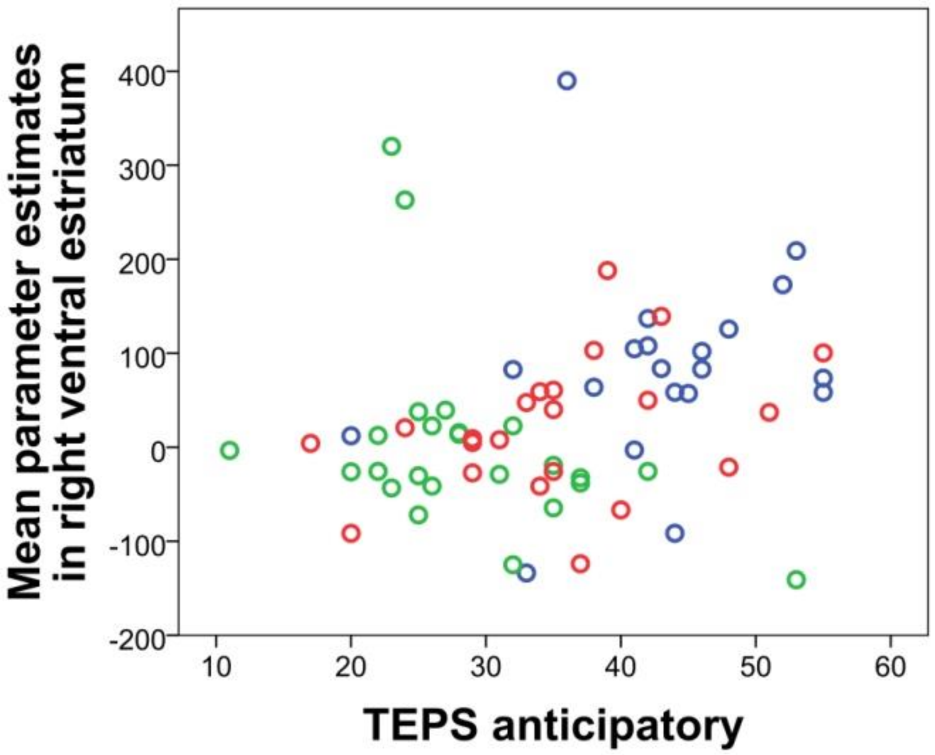

Supplement: Supplementary file 1 [file DataSheet1.PDF]
